# Identifying risk factors for the development of sepsis during adult severe malaria

**DOI:** 10.1186/s12936-018-2430-2

**Published:** 2018-07-31

**Authors:** Tsi Njim, Arjen Dondorp, Mavuto Mukaka, Eric O. Ohuma

**Affiliations:** 1 Centre for Tropical Medicine and Global Health, Nuffield Department of Medicine, University of Oxford, Old Road Campus, Oxford, OX3 7BN UK; 2 Health and Human Development Research Group, Douala, Cameroon; 3 Mahidol Oxford Tropical Medicine Research Unit, Faculty of Tropical Medicine, Mahidol University, 3/F, 60th Anniversary Chalermprakiat Building, 420/6 Rajvithi Road, Bangkok, 10400 Thailand; 4 Centre for Statistics in Medicine, Botnar Research Centre, Nuffield Department of Orthopaedics, Rheumatology and Musculoskeletal Sciences, University of Oxford, Windmill Road, Oxford, OX3 7LD UK

**Keywords:** Severe malaria, Sepsis, Prognostic model, Nomogram, Southeast Asia

## Abstract

**Background:**

Severe falciparum malaria can be compounded by bacterial sepsis, necessitating antibiotics in addition to anti-malarial treatment. The objective of this analysis was to develop a prognostic model to identify patients admitted with severe malaria at higher risk of developing bacterial sepsis.

**Methods:**

A retrospective data analysis using trial data from the South East Asian Quinine Artesunate Malaria Trial. Variables correlating with development of clinically defined sepsis were identified by univariable analysis, and subsequently included into a multivariable logistic regression model. Internal validation was performed by bootstrapping. Discrimination and goodness-of-fit were assessed using the area under the curve (AUC) and a calibration plot, respectively.

**Results:**

Of the 1187 adults with severe malaria, 86 (7.3%) developed clinical sepsis during admission. Predictors for developing sepsis were: female sex, high blood urea nitrogen, high plasma anion gap, respiratory distress, shock on admission, high parasitaemia, coma and jaundice. The AUC of the model was 0.789, signifying modest differentiation for identifying patients developing sepsis. The model was well-calibrated (Hosmer–Lemeshow Chi squared = 1.02). The 25th percentile of the distribution of risk scores among those who developed sepsis could identify a high-risk group with a sensitivity and specificity of 70.0 and 69.4%, respectively.

**Conclusions:**

The proposed model identifies patients with severe malaria at risk of developing clinical sepsis, potentially benefiting from antibiotic treatment in addition to anti-malarials. The model will need further evaluation with more strictly defined bacterial sepsis as outcome measure.

**Electronic supplementary material:**

The online version of this article (10.1186/s12936-018-2430-2) contains supplementary material, which is available to authorized users.

## Background

In the past decade, significant gains have been made in the global control of malaria with a decreased malaria incidence of 37.7% worldwide from 146 per 1000 to 91 per 1000 population [1, 2], and a 62% decrease in malaria-associated mortality in 2015 compared to 2000 [1–3]. However, malaria is still responsible for half a million deaths annually and the case fatality of severe malaria remains high. Patients with severe malaria are at increased risk of developing concomitant bacterial sepsis, which increases the risk of a fatal outcome. Recent studies in sub-Saharan Africa showed that 4–23% of children with severe malaria had concomitant sepsis [4–7]. In adult severe malaria, bacterial sepsis is less common but still considerable, with a reported incidence of 13% in Myanmar [8].

Although hypotension is much less common in severe malaria compared to bacterial sepsis [9], other clinical features of severe malaria and severe sepsis with organ failure overlap, hampering the diagnosis of concomitant sepsis. Blood cultures to diagnose invasive bacterial infections are rarely available in resource-limited settings, which comprises most of the malaria endemic countries [10]. In paediatric severe malaria, where concomitant bacterial sepsis is common, treatment guidelines recommend routine co-administration of antibiotics to anti-malarial treatment [11, 12]. Routine antibiotics it not recommended for adult patients with severe malaria. In this group it is important to identify patients at risk for concomitant bacterial sepsis, since these would require life-saving antibiotic treatment in addition to anti-malarial treatment. Antibiotics can then be withheld in patients identified at low risk for bacterial sepsis, saving costs and reducing antibiotic drug pressure driving antimicrobial drug resistance [13].

This analysis, therefore, aims to develop a prognostic model to identify adult patients with severe falciparum malaria at greater risk of developing sepsis using data from a large malaria treatment study.

## Methods

### Study design, study population and sampling

The study was a retrospective analysis of the South East Asian Quinine Artesunate Malaria Trial (SEAQUAMAT), a large randomized controlled trial (RCT) comparing intravenous artesunate with quinine as anti-malarial treatment. The SEAQUAMAT study was carried out from June 2003 to May 2005 in 10 healthcare facilities in four Southeast Asian countries: Bangladesh (n = 1), India (n = 1), Indonesia (n = 1) and Myanmar (n = 7). The study randomized 1461 patients with severe malaria into two arms, with 730 patients receiving artesunate and 731 patients receiving quinine. Inclusion and exclusion criteria, study procedures, and definitions for severe malaria have previously been published [14]. In addition, children (age ≤ 16 years, n = 274) were excluded from the present study. The main study outcome was in-hospital mortality from severe malaria. The study showed that in-hospital mortality was reduced by 34.7% in patients who received intravenous artesunate when compared to patients who received quinine arm [14]. Secondary outcomes included incidence of neurological sequelae and development of sepsis.

### Definitions of parameters

- Coma: Glasgow coma scale < 11/15 in adults.
- Clinical shock: Based on clinical judgement of admitting physician taking into consideration a low BP (< 80/60 mmHg) and cold extremities.
- Respiratory distress: Respiratory rate > 32 breaths per minute.
- Hyperparasitaemia: Asexual *Plasmodium falciparum* parasitaemia > 10%.
- Severe anaemia: Haemoglobin < 5.0 g/dl.
- Hypoglycaemia: Plasma glucose < 2.2 mmol/l.
- Acidosis: Blood bicarbonate < 15 mmol/l or base excess < 3.3 mmol/l.
- Acute kidney injury: Blood urea nitrogen > 20 mg/dl.
- Sepsis: The clinical diagnosis of sepsis was made by the attending physician based mainly on the development of shock (hypotension with cold extremities) as laboratory assessments such as full blood counts were not routinely available, and blood cultures were not performed.

### Statistical analysis

Means (standard deviations) or medians (interquartile range) were used to summarize continuous variables as appropriate while proportions and frequencies were used to summarize categorical variables. Variables potentially related with the development of sepsis were selected based on their association described in published literature and included: age; gender; presence of coma on admission; convulsions; prostration; shock (clinical shock and blood pressure); anaemia (haemoglobin and haematocrit levels); acute kidney injury [blood urea nitrogen (BUN) and serum potassium levels]; presence of jaundice; glycaemia levels; temperature; acidosis (Venous pH, base excess, total CO_2_, anion gap); parasite count and respiratory distress (respiratory rate and clinical respiratory distress).

A univariable logistic regression analysis was performed with sepsis as the outcome and all the potential predictors for development of sepsis in severe malaria. All variables with p values < 0.05 were included in the multivariable logistic regression analysis model. Subsequently, a bootstrap analysis with 5000 simulations was performed to compare the measures of effect obtained from the original model with the bootstrapped model. Variables which remained significant were then used to produce a nomogram.

Discrimination of the model was assessed by calculating the AUC of the ROC curve. Bootstrapping was done with 5000 simulations to obtain a bootstrapped AUC. This bootstrapped AUC was compared with the original AUC. The discriminative properties using the AUC were assessed using the following classification: 0.90–1 excellent; 0.80–0.90—good; 0.70–0.80—fair; 0.60–0.70—poor and 0.50–0.60—very poor discriminative properties [15]. Discrimination was further assessed using the Brier score and the Somers’ D coefficient. The Somers’ D obtained for the original model was compared to that obtained from applying the models from the bootstrapped datasets to the original dataset. An estimate of optimism was calculated as the difference between the two coefficients [16]. The optimism corrected estimate of the Somers’ D was then calculated and converted to a c-statistic to have an interpretation similar to the AUC.

Calibration was assessed by drawing a calibration plot after dividing the data into ten risk groups.

The predictors were then divided into categories with each category assigned a numerical value depending on the contribution of the predictor towards the probability of developing sepsis. For each patient, the numerical score of each predictor was added to produce a total score. The 25th percentile of the distribution of total scores among those with sepsis was used to classify patients at high risk of developing sepsis and respective sensitivity and specificity calculated.

A nomogram based on the numerical contributions of each of the predictors in the model was constructed from the logistic regression model. The data were analysed using the R statistical software package (R Foundation for Statistical Computing, Vienna, Austria) and Stata software package version 12 (Statacorp, College Station, TX, USA).

### Ethical considerations

The SEQUAMAT study received ethical clearance from OXTREC, the national ethical committees of the countries involved and from the ethical institutions of the four countries where the trial was conducted. Ethics and administrative approval was obtained from MORU (Mahidol-Oxford Tropical Research Unit) to use the data for this secondary data analysis. The analysis used de-identified data from the curated SEAQUAMAT database, which did not include patient names. Anonymized identification numbers were used.

## Results

A total of 1187 adults (age > 16 years) were included in the SEAQUAMAT trial and 899 (75.7%) were males. The ages ranged from 17 to 87 years with a median age of 28 (Interquartile range 22–40). More than half (52.6%) of the patients had jaundice on admission while 11.5% presented with shock (Table 1). A total of 86 (7.3%, 95% CI 5.8, 8.7) patients developed sepsis after admission. Indonesia had the highest proportion (19.2%) of patients who developed sepsis while Myanmar had the lowest (3.7%) (Table 1) (Additional file 1). Patients who developed sepsis were over three times more likely to die during admission than those who did not (OR: 3.48; 95% CI 2.15, 5.60; p value < 0.001).

**Table 1:** Baseline characteristics for patients admitted for severe malaria

	Characteristic	Category	N	%	Artesunate arm		Quinine arm		Sepsis development	
					n	(%)	n	(%)	n	(%)
1	Country	Myanmar	458	38.6	233	50.9	225	49.1	17	3.7
		Bangladesh	399	33.6	198	49.6	201	50.4	21	5.3
		India	137	11.4	67	48.9	70	51.1	11	8.0
		Indonesia	193	16.3	97	50.3	96	49.7	37	19.2
2	Sex	Male	899	75.7	456	50.7	443	49.3	48	5.3
		Female	288	24.3	139	48.3	149	51.7	38	13.2
3	Age	17–25	504	43.6	249	49.4	255	50.6	33	6.6
		26–35	321	22.2	167	52.0	154	48.0	26	8.1
		36–45	188	13.0	96	51.1	92	48.9	18	9.6
		46–87	174	12.1	83	47.7	91	52.3	9	5.2
4	Respiratory distress	Yes	117	9.9	51	43.6	66	56.4	20	17.1
		No	1070	90.1	544	50.8	526	49.2	66	6.2
5	Coma on admission	Yes	505	42.5	255	50.5	250	49.5	46	9.1
		No	682	57.5	340	49.9	342	50.2	40	5.9
6	Hypoglycaemia on admission (blood glucose < 2.2 mmol/l)	Yes	19	1.7	8	42.1	11	57.9	1	5.3
		No	1127	98.3	568	50.4	559	49.6	85	7.5
7	Shock on admission	Yes	137	11.5	65	47.5	72	52.6	21	15.3
		No	1050	88.5	530	50.5	520	49.5	65	6.2
8	Jaundice on admission	Yes	624	52.6	319	51.1	305	48.9	57	9.1
		No	563	47.4	276	49.0	287	51.0	29	5.2
9	Severe anaemia on admission (Hb < 5.0 g/dl)	Yes	60	5.0	31	51.7	29	48.3	11	18.3
		No	1127	95.0	564	50.0	563	50.0	75	6.7
10	Acute kidney injury (BUN > 20 mg/dl)	Yes	277	24.6	130	46.9	147	53.1	37	13.4
		No	850	75.4	440	51.8	410	48.2	45	5.3
11	Acidosis on admission (base excess < 3.3 mmol/l)	Yes	524	48.7	254	48.5	270	51.5	59	11.3
		No	551	51.3	289	52.5	262	47.5	23	4.2
12	Hyperparasitaemia on admission (> 10% RBCs infected)	Yes	185	15.6	99	53.5	86	46.5	26	14.1
		No	1002	84.4	496	49.5	506	50.5	60	6.0

*Hb* haemoglobin, *BUN* blood urea nitrogen, *RBC* red blood cells, *mmol/l* millimoles per litre, *g/dl* grams per decilitre, *mg/dl* milligrams per decilitre

### Model development

On univariable analysis, the following predictors were associated with development of sepsis: female sex (OR: 2.70; 95% CI 1.72, 4.22), jaundice on admission (OR: 1.85, 95% CI 1.17, 2.94), a high anion gap (OR: 1.04, 95% CI 1.01, 1.06), a high parasite count (OR: 1.04, 95% CI 1.02, 1.06), high blood urea nitrogen levels (OR: 1.01, 95% CI 1.01, 1.02), high respiratory rates on admission (OR: 1.03, 95% CI 1.01, 1.05), shock on admission (OR: 2.74, 95% CI 1.62, 4.65) and respiratory distress on admission (OR: 3.14, 95% CI 1.82, 5.39.74) (Table 2). Those who had lower mean arterial blood pressures (OR: 0.98, 95% CI 0.96, 0.99) and a low base excess (OR: 0.94, 95% CI 0.92, 0.97) were also more likely to develop sepsis (Table 2).

**Table 2:** Univariable analysis of predictors for the development of sepsis in patients admitted for severe malaria

	Characteristic	Category	N	OR	95% CI	p value
1	Age	Age (years)	1187	1.00	0.98, 1.01	0.749
2	Sex	Sex^a^				
		Female	288	0.37	0.24, 0.58	< 0.001
		Male	899	1.00		
3	Cerebral malaria	Coma (GCS < 11 or BCS < 3)				
		Yes	476	1.47	0.95, 2.28	0.088
		No	711	1.00		
		Convulsions				
		Yes	113	1.28	0.64, 2.54	0.490
		No	1074	1.00		
		Prostration^a^				
		Yes	435	0.61	0.37, 0.99	0.050
		No	752	1.00		
4	Haemodynamic shock	Systolic BP (mm/Hg)^a,b^	1177	0.98	0.97, 0.99	0.001
		Diastolic BP (mm/Hg)^a,b^	1176	0.98	0.96, 0.99	0.002
		MAP (mm/Hg)	1176	0.98	0.96, 0.99	0.001
		Clinical shock^a^				
		Yes	137	2.74	1.62, 4.65	< 0.001
		No	1050	1.00		
5	Severe anaemia	Haemoglobin (g/dl)^a^	1112	0.94	0.87, 1.00	0.052
		Haematocrit (%)	1100	0.98	0.96, 1.00	0.080
6	Renal failure	BUN level (mg/dl)^a^	1127	1.01	1.01, 1.01	< 0.001
		Serum potassium (mg/dl)	1076	1.23	0.94, 1.61	0.161
7	Jaundice	Jaundice^a^				
		Yes	624	1.85	1.17, 2.94	0.009
		No	563	1.00		
8	Hypoglycaemia	Glycaemia (mg/dl)	1146	1.00	0.99, 1.00	0.438
9	Hyperpyrexia	Temperature (°C)	1185	0.96	0.79, 1.16	0.655
10	Acidosis	Venous pH^a,b^	1068	0.09	0.02, 0.40	0.002
		Base excess (mmol/l)^a^	1075	0.94	0.92, 0.97	<0.001
		Total CO_2_^a,b^	1071	0.92	0.89, 0.96	< 0.001
		Anion gap (mmol/l)^a^	1050	1.04	1.01, 1.06	0.002
11	Hyperparasitaemia	Parasite count (/μl)^a^	1187	2.57	1.57, 4.19	< 0.001
		Percentage parasitaemia (%)^a,b^	1187	1.04	1.02, 1.06	< 0.001
12	Respiratory distress	Respiratory rate (cycles/min)^a^	1185	1.02	1.01, 1.05	0.003
		Respiratory distress^a^				
		Yes	117	3.14	1.82, 5.39	< 0.001
		No	1070	1.00		

*BCS* Blantyre coma score, *GCS* Glasgow coma score, *BP* blood pressure, *MAP* mean arterial blood pressure, *OR* odds ratio, *CI* confidence interval, *BUN* blood urea nitrogen, *mm/Hg* millimetres of mercury, *mmol/l* millimoles per litre, *g/dl* grams per decilitre, *mg/dl* milligrams per decilitre, *°C* degrees centigrade; *μl* microlitre, *min* minute

^a^Significant variables on univariate analysis

^b^Variables omitted from logistic regression model due to multicollinearity

On multivariable analysis after forward selection of variables, female sex (aOR: 2.36, 95% CI 1.34, 4.17), high blood urea nitrogen levels (aOR: 1.01, 95% CI 1.00, 1.02), high anion gap (aOR: 1.02, 95% CI 1.00, 1.05), high percentage parasitaemia (aOR: 1.03, 95% CI 1.01, 1.06), visible jaundice on admission (aOR: 1.78, 95% CI 1.03, 3.07), shock on admission (aOR: 2.85, 95% CI 1.35, 5.99), coma on admission (aOR: 2.17, 95% CI 1.21, 3.90) and respiratory distress on admission (aOR: 3.62, 95% CI 1.69, 7.76) remained statistically significant (Table 3). Results of the 5000 simulations by bootstrapping had the same measures of effect to those obtained by the logistic regression model (Table 3).

**Table 3:** Multivariable logistic regression and bootstrap analysis for predictors for development of sepsis in patients admitted for severe malaria

Predictors	Category	aOR	95% CI	p value	BOR	B 95% CI	B p value
Sex^*,b^	Female	2.36	1.34, 4.17	0.003	2.36	1.30, 4.29	0.005
	Male	1.00			1.00		
Coma on admission^b^	Yes	2.17	1.21, 3.90	0.010	2.17	1.19, 3.93	0.011
	No	1.00			1.00		
Prostration on admission	Yes	1.00	0.55, 1.84	0.989	1.00	0.55, 1.84	0.989
	No	1.00			1.00		
Mean arterial pressure (mmHg)		0.99	0.97, 1.01	0.325	0.99	0.97, 1.01	0.308
Shock on admission^b^	Yes	2.85	1.35, 5.99	0.006	2.85	1.29, 6.28	0.009
	No	1.00			1.00		
Haemoglobin on admission (mg/dl)		0.98	0.90, 1.07	0.646	0.98	0.89, 1.08	0.687
BUN (mg/dl)^*,b^		1.01	1.00, 1.02	0.004	1.01	1.00, 1.02	0.005
Visible jaundice on admission^b^	Yes	1.78	1.03, 3.07	0.038	1.78	1.02, 3.10	0.042
	No	1.00			1.00		
Base excess on admission (mmol/l)		1.01	0.97, 1.05	0.735	1.01	0.97, 1.05	0.742
Anion gap on admission^*,b^		1.02	1.01, 1.05	0.043	1.02	1.00, 1.05	0.050
Percentage parasitaemia on admission (%)^b^		1.03	1.01, 1.06	0.008	1.03	1.01, 1.06	0.014
Respiratory rate on admission (cycles/minute)		0.98	0.95, 1.02	0.344	0.98	0.95, 1.02	0.328
Respiratory distress on admission^b^	Yes	3.62	1.69, 7.76	0.013	3.62	1.64, 8.02	0.002
	No	1.00			1.00		
Somers’ D^a^		0.52			0.49		

*aOR* adjusted odds ratio, *CI* confidence interval, *BUN* blood urea nitrogen, *BOR* bootstrapped odds ratio, *B* bootstrapped, *mmol/l* millimoles per litre, *mg/dl* milligrams per decilitre

* Variables significant on multivariable analysis

^a^Estimate of optimism = 0.03

^b^Included in final regression model

### Model validation

**Fig. 1:**
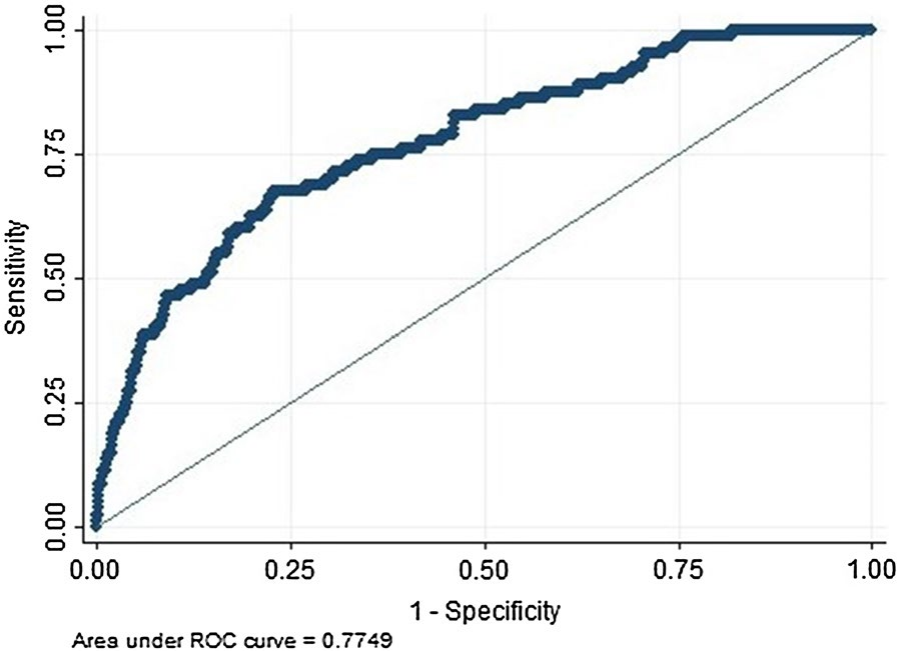
Receiver operating characteristic cure showing diagnostic accuracy of model to predict development of sepsis during admission for severe malaria

The AUC based on the logistic regression model was 0.78 (95% CI 0.72, 0.83) (Fig. 1). After 5000 simulations, the bootstrapped area under the curve was 0.78 (95% CI 0.72, 0.83). The Brier score obtained from the model was 0.06 (95% CI 0.05–0.07, p < 0.001). The optimism corrected estimate of the Somers’ D was 0.48 (Table 3) with a corresponding c-statistic of 0.74.

**Fig. 2:**
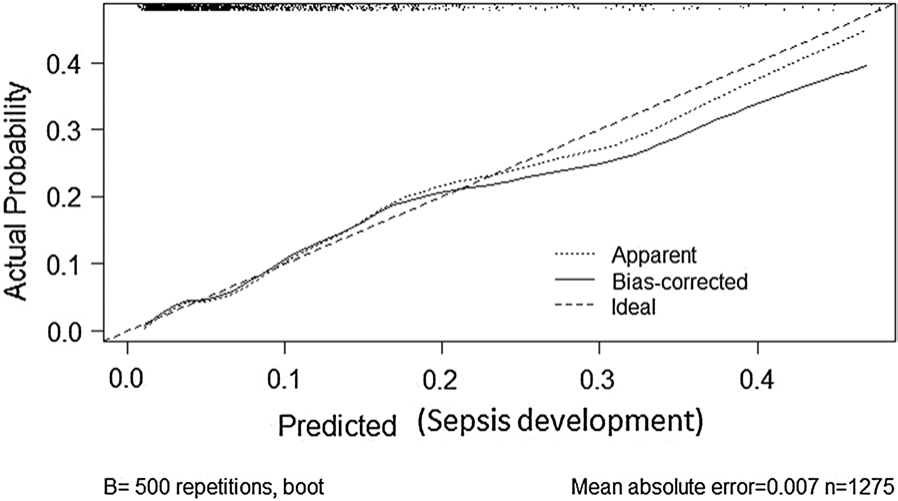
Graph showing a plot of the expected event probabilities against the predicted event probabilities with a perfect predictive ability shown on the graph by the diagonal red straight line at 45°

The participants were divided into ten groups based on the probability of developing sepsis. There was no difference between the observed values for patients who developed sepsis after admission for severe malaria with the values predicted by the model (Fig. 2, Hosmer–Lemeshow Chi squared statistic = 1.02, p = 0.995).

**Fig. 3:**
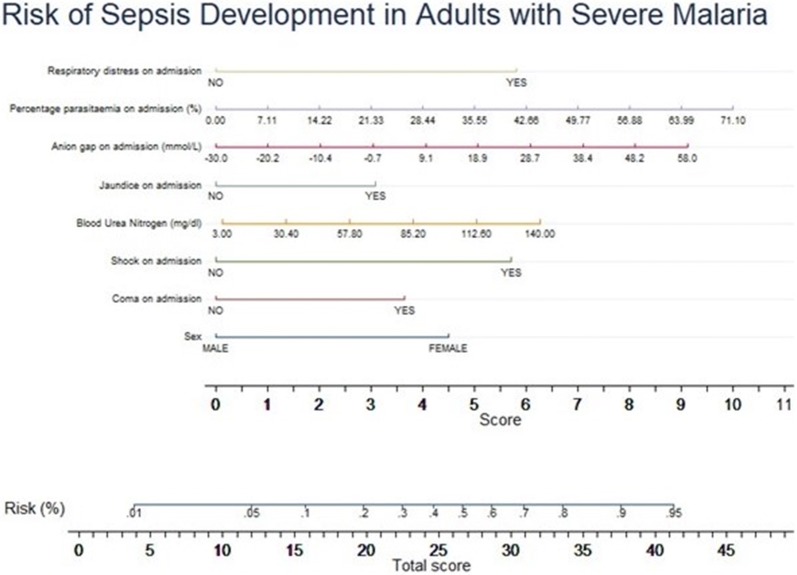
Nomogram showing the relative contributions of independent predictors to the development of sepsis of sepsis during admission for severe malaria

The predictors from the final model were arranged into categories with each category assigned a numerical value depending on its contribution to the development of sepsis (Table 4). The median, 25th and 75th percentile of the total score for those who developed sepsis were 18.8, 14.6 and 23.2 respectively. The median, 25th and 75th percentile of the total score for those who did not develop sepsis were 12.8, 9.9 and 16.5 respectively. The diagnostic properties of the model at cut-offs of the median, 25th and 75th percentile of the total score for those who developed sepsis were calculated (Table 5). A nomogram obtained from the above predictive variables is shown in Fig. 3.

**Table 4:** Numerical contributions of categories of predictors to development of sepsis

	Predictor	Percentage contribution of predictor (%)	Categories of predictor	Numerical contribution
1	Jaundice on admission	6.4	Yes	3.1
			No	0.0
2	Anion gap on admission	18.9	< − 30	0.0
			> − 30.0 to − 20.2	1.0
			> − 20.2 to − 10.4	2.0
			> − 10.4 to − 0.7	3.0
			> 0.7–9.1	4.1
			> 9.1–18.9	5.1
			> 18.9–28.7	6.1
			> 28.7–38.4	7.1
			> 38.4–48.2	8.1
			> 48.2–58	9.1
3	Percentage parasitaemia on admission	20.8	0	0
			0–7.1	1.0
			7.1–14.2	2.0
			14.2–21.3	3.0
			21.3–28.4	4.0
			28.4–35.6	5.0
			35.6–42.7	6.0
			42.6–49.8	7.0
			49.8–56.9	8.0
			56.9–64.0	9.0
			64.0–71.2	10.0
3	Sex	9.3	Male	0.0
			Female	4.5
4	Coma on admission	7.7	Yes	3.7
			No	0.0
4	Shock on admission	11.8	Yes	5.7
			No	0.0
5	Respiratory distress on admission	12.0	Yes	5.8
			No	0.0
6	Blood urea nitrogen levels on admission (mg/dl)	13.1	0.00–3.00	0.1
			> 3.00–30.4	1.4
			> 30.4–57.8	2.6
			> 57.8–85.2	3.8
			> 85.2–112.6	5.0
			> 112.6–140.0	6.3
7	Total possible score	100.0	–	48.2

**Table 5:** Diagnostic properties of model at various cut-off values of the total score

Cut-off	Total score	Prob (%)	Sepsis		No sepsis		Sensitivity	Specificity	PPV	NPV	OCCR
			PS	PNS	PS	PNS					
25th percentile	14.6	7.0	56	24	295	670	70.00	69.43	15.95	96.54	69.47
Median	18.8	18	31	49	63	902	38.75	93.47	32.98	94.85	89.28
75th percentile	23.2	31	15	65	21	944	18.75	97.82	41.67	93.56	91.77

*PS* predicted with sepsis, *PNS* predicted with no sepsis, *PPV* positive predictive value, *NPV* negative predictive value, *OCCR* overall correct classification rate, *Prob* probability

## Discussion

This study showed that the prevalence of patients who developed clinical sepsis after admission for severe malaria in Southeast Asia was 7.3%. It should be noted that severe sepsis in this study was defined clinically, mainly based on the development of shock (hypotension with cold extremities). Laboratory assessments such as full blood counts were not routinely available, and blood cultures were not performed. It is likely that the true incidence of bacterial sepsis complicating severe malaria was underestimated in this dataset. Indeed, the incidence of sepsis in this study was lower than the 13% incidence reported in a study from Myanmar in adult patients with severe malaria using blood culture results as an outcome measure [8].

In the current study, patients with malaria who developed clinical sepsis upon admission were three times more likely to die than patients who were admitted for severe malaria only. The increase in case fatality in patients with concomitant sepsis has also been described in African children with severe malaria. For instance, a study amongst 1780 children admitted with slide confirmed severe malaria in Mozambique showed that the case fatality rate rose from 4.0 to 22% in the presence of bacteraemia [12, 17]. Current WHO guidelines therefore recommend the co-administration of parenteral broad spectrum antibiotics in addition to parenteral artesunate for the treatment of severe malaria in children [11]. For adult patients with severe malaria routine antibiotics are not recommended, because of the lower incidence of concomitant bacterial sepsis. Identification of patients at risk of sepsis is thus important, since these patients will benefit from antibiotic treatment.

This model identified independent predictors for development of sepsis in severe malaria including female sex, coma on admission, respiratory distress on admission, high anion gap (acidosis), high parasitaemia, high BUN levels, shock on admission and visible jaundice on admission. All these predictors are clinical symptoms or laboratory parameters associated with both bacterial sepsis and severe malaria, except for patient gender. The finding that female patients were two times more likely to have concomitant sepsis than male patients was unexpected and unexplained. The finding is consistent with a Kenyan study on paediatric severe malaria, which also showed that bacteraemia occurred in significantly higher proportions in females than in males [18]. Coma is not a common finding in severe sepsis, unless there is severe septic shock or meningo-encephalitis as the source of sepsis. However, presence of coma predisposes for aspiration pneumonia, which can develop into sepsis.

Respiratory distress could result from acute respiratory distress syndrome (ARDS), metabolic acidosis, fluid overload, aspiration pneumonia or other concomitant diseases [19]. ARDS is a common complication of both sepsis and severe malaria. However, ARDS is more common in bacterial sepsis [18], which could partly explain its prognostic significance for sepsis in this study. Development of hospital-acquired pneumonia could contribute further to the development sepsis during admission [19]. Study findings are in line with a Kenyan study showing that although respiratory distress was common in malaria, it was more prominent in patients with a concomitant sepsis [18]. In African children with severe malaria, both *Streptococcus pneumoniae/Haemophilus influenzae* (causing mainly pneumonia) were shown to be a common cause of concomitant sepsis [5].

Metabolic acidosis in severe malaria mainly results from microvascular obstruction by infected erythrocytes compromising tissue perfusion, including in vital organs such as brain and kidney [19, 20], another complication found to be an independent predictor of development of sepsis in this study. Renal failure in sepsis involves immune-mediated microvascular and tubular dysfunction. Although the pathogenesis is different, metabolic acidosis and renal failure are also common complications in patients with severe sepsis [20, 21]. This could explain why acidosis and renal failure symptoms were predictive of concomitant sepsis in this analysis. Non-*Salmonella typhi* and gram negative bacteria (both gut bacteria) are known causes of sepsis in malaria [5]. It has been hypothesized that increased translocation of bacteria from the gut caused by the sequestration of parasitized red blood cells in the gut microcirculation are a causative factor [6, 22]. The latter is a function of the total parasite burden as are plasma PfHRP2 concentrations. This could thus link acidosis and high percentage of parasitaemia to the incidence of sepsis in malaria.

All four predictors: metabolic acidosis (measured as an increased anion gap), parasite burden (high parasitaemia), renal failure (measured as BUN levels) and hypotensive shock (by taking the blood pressure) are all parameters which can be used in resource-limited settings where laboratory facilities have been supplemented. The presented model could thus be beneficial in the field setting and aid clinicians to identify patients at risk for developing bacterial sepsis.

The AUC of the model (0.78) was similar to that of the validated bootstrapped AUC (0.78). The model fell within the range of fair discriminative properties (0.70–0.80). This was further confirmed using the Brier score and the Somers’ D. The Brier score from this model (0.06) is close to zero indicating that the model can distinguish between those who will not and those who will develop sepsis. The optimism corrected estimate of the Somers’ D obtained after bootstrapping (0.49) produced a c-statistic (0.74), which falls in the range of fair discriminative properties. The model was also properly calibrated as demonstrated in the calibration plot. There was no difference between the observed and predicted values for patients who developed sepsis.

Sepsis occurring in severe malaria is associated with an increased risk of mortality. A good model should have a reasonably high sensitivity to include a high number of individuals at risk of having the outcome. In this study, using a 7.0% probability had the highest sensitivity (70.0%). Though this sensitivity is modest, this prognostic model shows potential for predicting the development of sepsis in adult patients with severe malaria.

Nadjm et al. [10] produced a model to predict the risk of development of sepsis in severe malaria in 2010 limited to paediatric cases. This model had a high sensitivity (86%) but a low specificity (22.3%) [10]. The model also included several WHO criteria which were based on nonspecific symptoms limiting its application in clinical settings. One of the variables included in their research was the presence of HIV infection. HIV status was not assessed in the current study, but prevalence rate in this patient group was considered to be low. However, inclusion of this variable in future research may help improve the sensitivity of the model.

## Limitations

A limitation of the current study is that the clinical diagnosis for sepsis is not very accurate. Therefore, the study findings will have to be confirmed in future studies using more accurate diagnostic tools for diagnosing sepsis, including blood cultures or molecular methods. Blood cultures are usually considered to be the clinical gold standard for the diagnosis of sepsis, but their sensitivity is limited [23]. In addition, studies in different geographical regions with different epidemiological settings should confirm the generalizability of this study findings.

## Conclusions

The development of sepsis is relatively common in adults admitted with severe falciparum malaria. The identified prognosticators from the presented model identified patients with severe malaria at higher risk of developing clinical sepsis, potentially benefiting from antibiotic treatment in addition to anti-malarials. The model needs further evaluation with more strictly defined bacterial sepsis as outcome measure.

## Additional file

**Additional file 1.** Sociodemographic and clinical characteristics of 86 patients with a clinical diagnosis of sepsis among patients diagnosed with severe malaria.

## Abbreviations

WHO: World Health Organization
ICU: intensive care unit
RDT: rapid diagnostic test
SIRS: systemic inflammatory response syndrome
SEAQUAMAT: South East Asian Quinine Artesunate Malaria Trial
ROC: receiver operating characteristic
AUC: area under the curve
RCT: randomized control trial
BP: blood pressure
OXTREC: Oxford Tropical Research Ethics Committee
MORU: Mahidol Oxford Tropical Research unit
OR: odds ratio
CI: confidence interval
AOR: adjusted odds ratio
PS: predicted with sepsis
PNS: predicted with no sepsis
PPV: positive predictive value
NPV: negative predictive value
OCCR: overall correct classification
