# Supplementary material for: Identifying risk factors for the development of sepsis during adult severe malaria
Source: Malar J. 2018 Jul 31;17:278. doi: 10.1186/s12936-018-2430-2 (PMC6066934; doi:10.1186/s12936-018-2430-2)
Supplement: Supplementary file 1 — Additional file 1. Sociodemographic and clinical characteristics of 86 patients with a clinical diagnosis of sepsis among patients diagnosed with severe malaria. [file 12936_2018_2430_MOESM1_ESM.docx]

|  | Characteristic | Category | Sepsis development | |
| --- | --- | --- | --- | --- |
|  |  |  | n | (%) |
| 1 | Country | Myanmar | 17 | 19.8 |
|  |  | Bangladesh | 21 | 24.4 |
|  |  | India | 11 | 12.8 |
|  |  | Indonesia | 37 | 43.0 |
| 2 | Sex | Male | 59 | 55.8 |
|  |  | Female | 44 | 44.2 |
| 3 | Age | 17 – 25 | 33 | 38.4 |
|  |  | 26 – 35 | 26 | 30.2 |
|  |  | 36 – 45 | 18 | 20.9 |
|  |  | 46 – 87 | 9 | 10.5 |
| 4 | Respiratory distress | Yes | 20 | 23.3 |
|  |  | No | 66 | 76.7 |
| 5 | Coma on admission | Yes | 46 | 53.5 |
|  |  | No | 40 | 46.5 |
| 6 | Hypoglycaemia on admission (blood glucose <2.2mmol/L) | Yes | 1 | 1.2 |
|  |  | No | 85 | 98.8 |
| 7 | Shock on admission | Yes | 21 | 24.4 |
|  |  | No | 65 | 75.6 |
| 8 | Jaundice on admission | Yes | 57 | 66.3 |
|  |  | No | 29 | 33.7 |
| 9 | Severe anaemia on admission (Hb <5.0g/dl) | Yes | 11 | 12.8 |
|  |  | No | 75 | 87.2 |
| 10 | Acute kidney injury (BUN >20mg/dl) | Yes | 37 | 45.1 |
|  |  | No | 45 | 54.9 |
| 11 | Acidosis on admission (base excess <3·3 mmol/L) | Yes | 59 | 72.0 |
|  |  | No | 23 | 28.0 |
| 12 | Hyperparasitaemia on admission (>10% RBCs infected) | Yes | 26 | 30.2 |
|  |  | No | 60 | 69.8 |
